# Patient–provider communication about cigarette and e-cigarette use during pregnancy: Adaptation and validation of frequency and quality of communication measures among a sample of pregnant patients

**DOI:** 10.18332/tpc/193605

**Published:** 2024-10-07

**Authors:** Emily M. Richardson, Eric Schisler, Page D. Dobbs

**Affiliations:** 1 Eleanor Mann School of Nursing, University of Arkansas, Fayetteville, United States; 2 Department of Health, Human Performance and Recreation, University of Arkansas, Fayetteville, United States; 3 Center for Public Health and Technology, University of Arkansas, Fayetteville, United States; 4 Department of Healthcare Studies, Salem State University, Salem, United States; 5 College of Education and Health Professions Health, Human Performance and Recreation, University of Arkansas, Fayetteville, United States

**Keywords:** e-cigarette, cigarette, health communication, patient–provider communication, scale development

## Abstract

**INTRODUCTION:**

Quality of patient–provider communication regarding tobacco use may encourage cessation that could lead to improved health outcomes for mothers and children. However, currently there are no validated measures of frequency and quality of patient–provider communication about cigarettes and e-cigarettes. The objective of this study was to adapt and validate measures of frequency and quality of patient–provider communication about smoking and e-cigarette use among a sample of pregnant mothers who currently smoked.

**METHODS:**

An online sample of US pregnant women who reported past 30-day smoking were recruited to complete a cross-sectional, online survey (n=267). An exploratory factor analysis examined the factor structure of four measures of frequency and quality of patient–provider communication about cigarettes and e-cigarettes among those who reported prior communication with their provider about cigarettes and e-cigarettes (n=170). Relationships between measures were explored, and a logistic regression explored each measure’s association with intention to switch from cigarettes to e-cigarettes.

**RESULTS:**

Items measuring the frequency of communication loaded onto one factor for both cigarettes and e-cigarettes (α=0.88). Quality of communication loaded onto two factors for both cigarettes and e-cigarettes, termed active communication and internalized perception. Internalized perceptions of communication quality about cigarettes (β= -0.32, p<0.002), active communication (β=0.46, p<0.02), and internalized perceptions of communication about e-cigarettes (β= -0.36, p<0.001) were related to intention to switch, in separated models.

**CONCLUSIONS:**

Quality conversations between healthcare providers and pregnant patients is likely more important for behavioral decision-making than the frequency of communication.

## INTRODUCTION

Using tobacco products during pregnancy can have generational effects of morbidity and mortality^1^. While it is well established that smoking during pregnancy is harmful, evidence suggests that nicotine use during pregnancy may be associated with long-term health effects for the child, including impaired fertility, obesity, hypertension, neurobehavioral deficiency, and respiratory disfunction^1,2^. Further, use of electronic cigarettes (e-cigarettes), which often contain high amounts of nicotine^3^, have been associated with higher rates of negative health outcomes, such as pre-term birth and small-for-gestational-age infants when compared to non-e-cigarette users^4^. Despite this, pregnant women continue to smoke cigarettes, with some switching to e-cigarettes due to comparative perceived safety^5^; e-cigarettes are the most common source of non-cigarette tobacco among pregnant women^6^. This comparative perceived safety is often referred to as a harm reduction approach, and past research suggests male physicians may be more likely to endorse this approach when communicating with patients who smoke than female physicans^7^.

Approximately 22% of physicians report recommending e-cigarettes as a cessation tool^8^, despite mixed evidence of their efficacy and safety^9,10^. Researchers caution pregnant women from using e-cigarettes as a cessation device due to potential for adverse gestational health outcomes^11,12^. Alternatively, few healthcare providers screen for e-cigarette than cigarette use, particularly among women of gestational age^13^. Despite the established need for quality patient–provider communication about smoking and e-cigarettes^14^, little literature has examined communication between providers and pregnant patients about e-cigarettes^13^, with no studies found that assessed communication quality about cigarettes and e-cigarettes among pregnant patients. In response, there has been a call for more research regarding the communication of comparative risks of e-cigarettes between healthcare providers and pregnant patients^15^.

Existing literature about patient–provider communication regarding cigarettes has focused on the comprehensiveness and communication skills of those delivering cessation^16^, factors associated with implementing the 5As (ask, advise, assess, assist, arrange) behavioral intervention method among pregnant women^17^, and using communication of personalized disease risk to motivate smoking cessation^18^. One study has examined the relationship between patient–provider communication and e-cigarette perceived harmfulness; however, the communication quality measure used broad language ‘about their healthcare’ and was not adapted to ask specifically about communication the patients had with their provider about cigarettes and e-cigarettes^14^. In lieu of this dearth of literature, we broadly explored measures regarding smoking communication validated in other populations and found a PhenX Tool kit measure created to assess the frequency and quality between parents and their children about smoking^19^. Using this instrument, we sought to adapt and validate a measure regarding the frequency and quality of patient–provider communication about cigarette and e-cigarette use during pregnancy. We also examined the relationship between each of these communication measures and pregnant mothers’ intention to switch from cigarettes to e-cigarettes.

## METHODS

### Participants and procedures

Between November and December 2019, a sample of pregnant women (n=267), aged 18–40 years who resided in the US and had smoked at least one cigarette in the past 30 days, were collected via Dynata, an online, third-party paneling service. Using this third-party paneling service, we recruited participants deemed difficult to reach – currently pregnant and self-reported past 30-day use of combustible cigarettes. Incentives such as cash and gift cards were provided by Dynata for participation. More information regarding the sampling procedures have been published elsewhere^20^. The Institutional Review Board of the University of Arkansas approved all procedures.

### Measures

Participant demographics collected included race/ethnicity, annual household income, education level, age, and relationship status. Cigarette and e-cigarette use was measured by asking participants: ‘Have you used regular cigarettes?’ and ‘Have you used e-cigarettes?’ with responses ‘Yes, in the past 30 days’, ‘Yes, in the past year, but not in the past 30 days’, and ‘Yes, but not in the past year/No, I have not’. Groups were categorized into current (past 30-day use) use, ever use, and never use for both cigarette and e-cigarette users. To specify that e-cigarette behaviors were specific to nicotine and not delta-9-tetrahydrocannabinol (THC), the following definition of e-cigarettes was included above all questions: Electronic cigarettes and other electronic vaping products include electronic hookahs (e-hookahs), vape pens, e-cigars, and products that are battery powered, usually contain nicotine, and flavors such as fruit, mint, or candy.

This study used a standard measure, freely available from the PhenX ToolKit, (http://www.phenxtoolkit.org) that originally assessed smoking-specific communication quality (protocol 710101) and frequency (protocol 710201) between mothers and their adolescent children^19^. For the purposes of this study, the measure survey was adapted to be more appropriate for the patient–provider relationship than between parents and children. Both tools have high validity in multiple other studies, are easily accessible, and require little effort to complete which made them ideal for adaptation in this population^19^.

*Frequency of smoking-specific communication*

Frequency questions included five items that asked about providers’ discouragement of cigarette use since the client became pregnant and how often their provider educated on adverse fetal outcomes and potential pregnancy complications related to cigarette use while pregnant with statements such as: ‘Since you became pregnant, how many times has your provider encouraged you to refrain from smoking cigarettes while pregnant?’^19^. Participants responded using a 5-point Likert scale ranging from never (1) to very often (5). Prior research found strong internal reliability (α=0.86-0.91) among different subgroups of adolescents^21^.

*Quality of smoking-specific communication*

Quality of communication assessed the client’s perspective of mutual respect and rapport with their provider while discussing the use of cigarettes during pregnancy. This six-item measure (adapted from an original eight-item measure) asked about comfort levels discussing cigarette use during pregnancy, ease of conversation, and trustworthiness with statements such as: ‘My healthcare provider and I are interested in each other’s opinions about smoking cigarettes during pregnancy’19. Two items were removed due to their inability to translate from the parent–child to the patient–provider relationship. An example of removed items includes: ‘How often does your parent talk about punishment if you smoked?’. Responses were measured using a 5-point Likert scale, ranging from completely not true (1) to completely true (5). Two negatively worded items, ‘When talking about smoking cigarettes during pregnancy I think my healthcare provider is dishonest/unreasonable’ were recoded so that all items were anchored in a positive direction. Prior studies report strong internal reliability of this measure (α=0.74–0.88) across waves and adolescent populations^21^.

*Frequency of e-cigarette-specific communication*

Similar to frequency of communication about smoking, this five-item measure was adapted to ask questions such as how often the provider had discouraged e-cigarette use since the client became pregnant. Participants responded using a 5-point Likert scale ranging from never (1) to very often (5).

*Quality of e-cigarette-specific communication*

Like quality of smoke-specific communication, these six items measured the quality of communication between the provider and client. Responses included statements such as: ‘My healthcare provider and I are interested in each other’s opinions about using e-cigarettes during pregnancy’. Participants provided responses using a 5-point Likert scale ranging from completely not true (1) to completely true (5). Two negatively worded items were recoded.

*Intention to switch from cigarettes to e-cigarettes*

Intention to switch to e-cigarettes was measured with the single statement: ‘I intend to switch from using cigarettes to e-cigarettes throughout the remainder of my pregnancy’. This single item was taken from a four-item scale previously used to measure intention to switch from cigarettes to e-cigarettes^20^. Responses were measured using a 7-point Likert scale ranging from strongly disagree (1) to strongly agree (7).

### Data analysis

SPSS 27 software was used to analyze all data. The original sample included 267 participants. Overall, 244 (91.4%) and 175 (65.5%) of the participants indicated that their provider had asked about their smoking and e-cigarette use, respectively. Thus, the final sample (n=170) included only those with prior communication with their provider about both cigarettes and e-cigarettes. After removing cases with missing responses to at least one communication measure (n=5), the final sample included 170 participants. The sample was determined to provide sufficient power based on standards that the variable-to-factor ratio should exceed six^22^.

We used an exploratory factor analysis to examine the factor structure of each scale (frequency/quality of communication about smoking, and frequency/quality of communication about e-cigarette use). Given that all items from each of the four measures were evenly distributed, we used a principals axis factoring extraction method with an oblique rotation, recommended for simple structures and accounts for relationships between factors^23^. Factor loadings were required to correlate with a single factor at ≥0.40 and cross-loading was conservatively considered when the item loaded with another factor >0.10. Lastly, we checked reliability using Cronbach’s alpha and examined the relationship between the communication scales (using Pearsons’ r)^24^. We also examined the relationship between the participants’ intention to switch from cigarettes to e-cigarettes and each of the communication scales and sub-scales using a linear regression, reporting the beta coefficient (β), standard error (SE), and p-value. We interpreted the variability using the adjusted R^2^.

## RESULTS

The sample consisted of pregnant women who had ever used cigarettes aged 18–40 years, where the average age was 29.1 years (SD=5.5). Overall, 45.9% (n=78) of participants identified as non-Hispanic White, 21.2% (n=36) as non-Hispanic Black or African American, and 11.8% (20) as Hispanic. About one-third (33.5%, n=57) had completed a high school or general education diploma, and a majority of the participants (58.2%, n=99) were married or in a domestic partnership. When asked about e-cigarette use, 53.5% report use in the past 30 days. See Table 1 for full demographic information.

**Table 1:** Demographic information of pregnant women who smoked and talked to their healthcare provider about cigarettes and e-cigarettes in the past 30-days, cross-sectional survey from 2019 (N=170)

*Characteristics*	*n (%)*
**Age** (years), mean (SD)	29.1 (5.5)
Race/ethnicity*	
Non-Hispanic White	78 (45.9)
Non-Hispanic Black or African American	36 (21.2)
Hispanic	20 (11.8)
Non-Hispanic Asian	10 (5.9)
Non-Hispanic American Indian or Alaskan Native	5 (2.9)
More than one race/ethnicity	20 (11.8)
**Education level**	
Lower than high school	7 (4.1)
High school or general education diploma (GED)	57 (33.5)
Associates degree or some college	32 (18.8)
Bachelor’s degree	37 (21.8)
Graduate or professional degree	37 (21.8)
**Annual household income** ($)	
<20000	30 (17.6)
20000–39999	45 (26.5)
40000–59999	32 (18.8)
60000–99999	30 (17.6)
>100000	33 (19.4)
**Marital status**	
Single/never married	66 (38.8)
Married/domestic partnership	99 (58.2)
Widowed/divorced/separated	5 (2.9)
**E-cigarette use**	
Ever use	155 (91.2)
Past 30-day use	91 (53.5)

Age limited to 18 to 40 years.

* Indicates 1 missing response.

### Frequency of smoking-specific communication

As expected, the five-item frequency of smoking-specific communication scale loaded onto a one-factor solution (loadings ranged from 0.73 to 0.84). We found this measure to demonstrate strong reliability (α=0.89), and it explained 62.6% of the variability about communication frequency between pregnant women and then healthcare provider when discussing smoking. See Table 2 for full factor loadings for the frequency and quality of smoking specific communication scales.

**Table 2:** Healthcare provider frequency and quality of communication with pregnant women about smoking during pregnancy, a cross-sectional survey from 2019 (N=170)

	*Item descriptive statistics*			*Factor loadings*		
	*Mean (SD)*	*Skewness*	*Kurtosis*	*Comm. frequency*	*Active Comm.*	*Internalized perception of Comm.*
**Frequency of smoking-specific communication**						
**Since you became pregnant, how many times has your healthcare provider:**						
Encouraged you to refrain from smoking cigarettes while pregnant?	3.96 (1.12)	-0.86	-0.09	0.80		
Discouraged you from being around friends who smoke cigarettes?	3.65 (1.23)	-0.61	-0.57	0.73		
Discussed the dangers of smoking cigarettes while pregnant with you?	3.72 (1.23)	-0.59	-0.67	0.84		
Talked about potential health outcomes that could occur to your baby if you smoke cigarettes while pregnant?	3.76 (1.25)	-0.62	-0.75	0.79		
Told you not to smoke cigarettes while pregnant?	3.79 (1.20)	-0.70	-0.41	0.80		
**Quality of smoking-specific communication**						
My healthcare provider and I are interested in each other’s opinions about smoking cigarettes during pregnancy.	3.78 (1.17)	-0.65	-0.38		0.53	-0.17
My healthcare provider and I are able to talk easily about our opinions concerning smoking cigarettes during pregnancy.	3.85 (1.04)	-0.70	0.15		0.77	0.03
When my healthcare provider and I are talking about smoking cigarettes during pregnancy we both feel comfortable.	3.75 (1.07)	-0.56	-0.26		0.85	0.03
When talking about smoking cigarettes during pregnancy I think my healthcare provider is dishonest.^§^	2.88 (1.46)	0.21	-1.32		0.07	0.94
When talking about smoking cigarettes during pregnancy I think my healthcare provider is unreasonable.^§^	3.03 (1.51)	0.03	-1.45		-0.08	0.79
Whenever my healthcare provider and I discuss smoking cigarettes during pregnancy I feel he/she takes me seriously.	3.90 (1.06)	-0.66	-0.43		0.75	0.06
Cronbach’s α				0.89	0.81	0.85

Comm.: communication.

§ Item reverse coded.

### Quality of smoking-specific communication

Instead of a one-factor model, we found the six-items quality of smoking-specific communication scale loaded onto two separate factors. Factor loading ranged from 0.53 to 0.85 for the first factor (active communication) and 0.79 to 0.94 for the second factor (internalized perception of communication). When checking the reliability, we confirmed that the Cronbach’s alpha was weaker (α=0.53) when all factors were loaded onto a one-factor model than when we loaded onto two separate factors. The first factor included four items that measured the active communication between the provider and the client (e.g. ease of communication; α=0.81). However, the two reverse coded items that measured participants perception if their healthcare provider was dishonest and unreasonable when talking about smoking, loaded onto a second factor (internalized perception; α=0.85). This two-factor model explained 61.7% of the variability about the quality of communication between providers and pregnant women when discussing smoking during pregnancy.

### Frequency e-cigarette-specific communication

Similar to the smoke-specific scale, the five-item quality of e-cigarette-specific communication scale loaded strongly onto a single factor solution (loadings ranged from 0.73 to 0.80). These five items about the frequency of communication between providers and pregnant women about e-cigarette use were found to have strong reliability (α=0.88), and they explained 59.1% of the variability about this communication (Table 3).

**Table 3:** Healthcare providers frequency and quality of communication with pregnant women about e-cigarette use during pregnancy, a cross-sectional survey from 2019 (N=170)

	*Item descriptive statistics*			*Factor loadings*		
	*Mean (SD)*	*Skewness*	*Kurtosis*	*Comm. frequency*	*Active Comm.*	*Internalized perception of Comm.*
**Frequency of e-cigarette-specific communication**						
**Since you became pregnant, how many times has your healthcare provider:**						
Encouraged you to refrain from using e-cigarettes while pregnant?	4.31 (1.61)	-0.32	-1.11	0.76		
Discouraged you from being around friends who use e-cigarettes?	3.95 (1.65)	-0.11	-1.07	0.73		
Discussed the dangers of using e-cigarettes while pregnant with you?	3.98 (1.60)	-0.04	-1.11	0.80		
Talked about potential health outcomes that could occur to your baby if you use e-cigarettes while pregnant?	3.96 (1.70)	-0.10	-1.20	0.78		
Told you not to use e-cigarettes while pregnant?	4.11 (1.69)	-0.21	-1.24	0.78		
**Quality of e-cigarette-specific communication**						
My healthcare provider and I are interested in each other’s opinions about using e-cigarettes during pregnancy.	3.88 (1.16)	-0.92	0.19		0.65	-0.18
My healthcare provider and I are able to talk easily about our opinions concerning using e-cigarettes during pregnancy.	3.95 (1.05)	-0.93	0.47		0.77	0.05
When my healthcare provider and I are talking about using e-cigarettes during pregnancy we both feel comfortable.	3.84 (1.05)	-0.62	-0.19		0.68	-0.03
When talking about using e-cigarettes during pregnancy I think my healthcare provider is dishonest.^§^	2.88 (1.50)	0.150	-1.41		-0.07	0.83
When talking about using e-cigarettes during pregnancy I think my healthcare provider is unreasonable.^§^	2.85 (1.52)	0.21	-1.42		0.06	0.94
Whenever my healthcare provider and I discuss using e-cigarettes during pregnancy I feel he/she takes me seriously.	3.87 (1.11)	-0.81	0.06		0.77	0.09
Cronbach’s α				0.88	0.81	0.88

Comm.: communication.

§ Item reverse coded.

### Quality of e-cigarette-specific communication

Similar to the quality of e-cigarette-specific communication scale, we found the quality of e-cigarette-specific communication scale to load onto two separate factors. This was confirmed with stronger internal reliability for a two-factor model (α=0.81 and α=0.88 for the active communication and communication internalized perception, respectively) than a one-factor model (α=0.45). Again, the two reverse coded items loaded separately (factor loadings were 0.83 and 0.94), representing the participants internalized perception about the communication. The remaining four items measured active communication (factor loadings ranged from 0.65 to 0.77). This six-item measure explained 61.8% of the variability of communication quality about e-cigarettes between a pregnant patient and their healthcare provider.

### Correlations between communication measures

All scales were significantly related to one another. The strongest relationships were between the cigarette and e-cigarette scale equivalents. For example, the frequency of smoking-specific communication scale and the frequency of e-cigarette-specific communication scales were strongly correlated (r=0.64, p<0.001). Additionally, quality of smoking-specific communication – active communication and the quality of e-cigarette-specific communication – active communication scales were strongly correlated (r=0.79, p<0.001). Notably, both internalized perceptions of communication scales were inversely related to all scales except their scale equivalent or cigarette and e-cigarette, suggesting that increased frequencies and active communication with the patient may be inversely associated with the patients’ internalized perceptions of the content shared with them. See Table 4 for the correlation matrix between all factors.

**Table 4:** Correlation matrix between communication measures, a cross-sectional survey from 2019 (N=170)

	*FSSC*	*QSSC-AC*	*QSSC-IP*	*FESC*	*QESC-AC*	*QESC-IP*
**FSSC**	1					
**QSSC-AC**	0.65***	1				
**QSSC-IP**	-0.16*	-0.23**	1			
**FESC**	0.64***	-0.26***	0.52***	1		
**QESC-AC**	0.64***	0.79***	-0.22**	0.57***	1	
**QESC-IP**	-0.18*	-0.18*	0.78***	-0.32***	-0.27***	1

FSSC: frequency of smoking-specific communication. QSSC-AC: quality of smoking-specific communication – active communication. QSSC-IP: quality of smoking-specific communication – internalized perception of communication. FESC: frequency of e-cigarette-specific communication. QESC-AC: quality of e-cigarette-specific communication – active communication. QESC-IP: quality of e-cigarette-specific communication – internalized perception of communication.

* p<0.05.

** p<0.01.

*** p<0.001.

### Communication and intention to switch to e-cigarettes

First, we measured the unique contribution of smoking-specific communication frequency, active communication, and internalized perception of communication on participants’ intention to switch to e-cigarettes. We found internalized perception of communication to be inversely related (β= -0.322, p=0.002) to intention to switch. These three factors explained 9% of the variability of intention to switch. In the second model, we measured the unique contribution of e-cigarette-specific communication (frequency, active communication, and internalized perception) on the participants’ intention to switch. We found both active communication (β=0.46, p=0.020) and internalized perception (β= -0.36, p<0.001) were associated with intention to switch. When all variables were combined into a single model, no sub-scale was significantly associated with intention to switch, likely due to the multicollinearity between items. See Table 5 for full model results.

**Table 5:** Communication and intention to switch to e-cigarette use during pregnancy, a cross-sectional survey from 2019 (N=170)

	*Model 1 (smoking-specific)*			*Model 2 (e-cigarette-specific)*			*Model 3 (both scales)*		
	*β*	*SE*	*p*	*β*	*SE*	*p*	*β*	*SE*	*p*
FSSC	0.16	0.18	0.399				0.21	0.21	0.314
QSSC-AC	0.26	0.22	0.236				0.20	0.28	0.490
QSSC-IP	-0.32	0.10	0.002				-0.11	0.17	0.500
FESC				-0.11	0.81	0.394	-0.19	0.14	0.183
QESC-AC				0.46	0.20	0.020	0.21	0.28	0.448
QESC-IP				-0.36	0.10	0.001	-0.29	0.17	0.085

FSSC: frequency of smoking-specific communication. QSSC-AC: quality of smoking-specific communication – active communication. QSSC-IP: quality of smoking-specific communication – internalized perception of communication. FESC: frequency of e-cigarette-specific communication. QESC-AC: quality of e-cigarette-specific communication – active communication. QESC-IP: quality of e-cigarette-specific communication – internalized perception of communication.

## DISCUSSION

The current study adapted and validated a measure regarding the frequency and quality of patient–provider communication about cigarette and e-cigarette use during pregnancy. Using these validated measures, we were able to determine associations between these communication scales and pregnant women’s intention to switch from cigarettes to e-cigarettes during pregnancy. It is well established that patient–provider education about health decision-making is important^25^, especially for cigarette use among pregnant patients^17^. Further, past research has emphasized the need for primary data regarding patient–provider communication specific to cigarettes and e-cigarettes^14^. Our findings provide a validated measure regarding quality of pregnant patient–provider communication, which have been far less studied than other patient populations^14^. The two unique constructs of communication quality indicate that communication perceived to be honest and reasonable (internalized perception) may be fundamentally different than patients’ perception of their healthcare providers’ interest or ease of conversation (active communication). When making decisions about cigarette and e-cigarette use during pregnancy, our findings suggest that the authenticity of healthcare providers’ communication is more important than the frequency of these conversations.

Across all three regression models, frequency was not associated with intention to switch. Certainly, quitting all tobacco products is the safest alternative for both the mother and developing fetus^5^; however, healthcare providers who report uncertainty about the harms and benefits of e-cigarette use may recommend switching to e-cigarettes, be unsure how to discuss e-cigarette use, or be unsure what advice to give those who request information^8^. At the time these data were collected, deaths resulting from e-cigarette or vaping-associated lung injury (EVALI) were eliciting broad public awareness to the harms of e-cigarette use, particularly among youth and pregnant women^26^. Although these deaths were found to be attributed to the areolation of vitamin E acetate, a chemical used with cannabis-containing vaping devices^26^, communication between pregnant women and healthcare providers about e-cigarette use during this timeframe was encouraged, and the Centers for Disease Control and Prevention discouraged use among pregnant patients^27^. Prior to these events, many providers report rarely asking participants about e-cigarette use, citing uncertainty of long-term risks as a limiting factor in communication^8^. However, pregnant women included in our sample may have been exposed to more communication about e-cigarettes than prior samples.

Patients internalized perception of communication with their healthcare provider about cigarettes and e-cigarettes may impact their health decision-making process. Pregnant women are 1.4 times more likely to visit a healthcare provider compared to non-pregnant women^28^, making pregnancy a key time when providers have a captive audience to inquire and educate about potential or known risks associated with e-cigarette use, including negative pregnancy and fetal outcomes. Current strategies encouraged for providers among pregnant women include using the 5As to encourage cessation at this pivotal time^17^, where nicotine exposure could be especially influential on brain development^29^. Prior research suggests intention to quit smoking may not be influenced by other’s condoning the behavior but rather providers presenting convincing evidence and empathetic support to quit^20^. Thus, application of the measures adapted in this study could be used to evaluate if intervention strategies improve providers’ communication with participants. Although some suggest that switching to e-cigarette during pregnancy may be a viable option for those who cannot quit smoking^30^, others raise cautions for providers to recommend this during pregnancy due to the lack of long-term evidence of health outcomes^12^. As providers consider communication information with their pregnant patients about e-cigarettes, they should consider where they receive their information and the credibility of the source.

In 2023, Medscapes, a leading medical information company, provided Continuing Medical Education (CME) hours for attendance of virtual educational courses sponsored by Philip Morris International (PMI)^31^. This education touted e-cigarettes as a viable cessation option, failing to recognize that most smokers who become dual users (use both cigarettes and e-cigarettes) fail to quit smoking exclusively^32^. Industry interference with health education for healthcare providers may impact patient–provider communication and long-term health outcomes, particularly for pregnant patients. For example, providers that recommend switching rather than quitting would expose a pregnant patient and fetus to nicotine throughout gestation, which has been linked to adverse birth outcomes^11^. Further, if different providers (e.g. obstetricians and gynecologist [OBGYNs] and primary care providers) share different information with the same patient, this could cause confusion or mistrust between patients and their providers.

### Limitations

Limitations of the study include the convenience sampling method employed, the online collection of responses, and the fact that the survey was only provided in English. Therefore, our population was limited to those with internet access, self-reported as pregnant, and English proficient. Data regarding number of pregnancies, gestational age, type of healthcare provider to whom they spoke, or initiation/frequency of prenatal visits were not collected, which limit information about the pregnant patients’ experiences and communication. Limitations with cross-sectional, self-reported data prohibit any causality claims from being assumed with our findings. Provided that our sample was deemed hard to reach, findings may be subject to social desirability bias, where pregnant women would be hesitant to disclose cigarette or e-cigarette use during pregnancy or adverse perceptions of communication with providers. Externally, the attention from EVALI related deaths at time of data collection may have created historical bias that influenced the perceived risk of participants. Multicollinearity between communication measures exist in the linear regression models. Finally, we did not ask participants about their intention to completely quit all nicotine-containing products that would have provided additional insight.

## CONCLUSIONS

Patients’ internalized perception of the conversations they have with their providers is an important communication construct when examining quality of patient–provider communication and health decision-making about use of cigarettes or e-cigarettes during pregnancy. Our findings suggest that the patients’ perception of the conversation may be related to their health decisions more than the frequency of which their provider asks about their use. Thus, instead of encouraging providers to ask about use, we may also need to encourage providers to engage in meaningful conversations with their patients about the harms of nicotine and tobacco use during pregnancy. This may mean that providers, particularly those who see pregnant patients (i.e. OBGYNs) need additional training about the harms of e-cigarette use during pregnancy and communication techniques for helping to talk to pregnant patients who use cigarettes and/ or e-cigarettes. Ultimately, healthcare providers offer a unique and important contribution to smoking cessation, particularly among pregnant patients, and by validating measures of communication quality and frequency, we can better understand the impact that patient–provider communication has on gestational cigarette and e-cigarette use.

## Data Availability

The data supporting this research are available from the authors on reasonable request.
